# Perceived parental alcohol problems and psychosomatic complaints among adolescents in Sweden

**DOI:** 10.1016/j.abrep.2023.100491

**Published:** 2023-04-24

**Authors:** Numan Raza Syed, Joakim Wahlström, Sara Brolin Låftman, Johan Svensson

**Affiliations:** a Department of Public Health Sciences, Stockholm University, Albanovägen 12, 106 91 Stockholm, Sweden; b The Swedish Council for Information on Alcohol and Other Drugs (CAN), Östgötagatan 90, 116 64 Stockholm, Sweden

**Keywords:** Adolescents, Alcohol, Parental drinking problems, CAST-6, Psychosomatic complaints

## Abstract

**Introduction:**

For adolescents, parental problem drinking can be regarded as a chronic stressor, negatively affecting their health. There is limited knowledge and a relative lack of empirical evidence on this topic, especially in Sweden. The aim of the current study was to examine perceived parental alcohol problems and the links with psychosomatic complaints among adolescents in Sweden.

**Methods:**

Data were obtained from the Swedish Council for Information on Alcohol and Other Drugs’ national survey of 2021, collected amongst 9,032 students in grades 9 (∼15–16 years) and 11 (∼17–18 years). Perceived parental alcohol problems were measured by the Children of Alcoholics Screening Test (CAST-6) scale, using a cutoff at ≥ 3. Psychosomatic complaints were captured by a binary measure based on the frequency of headache, stomach ache, feeling depressed or down, difficulties to fall asleep, and sleeping poorly at night. Sociodemographic characteristics included gender, grade, parental education, and parental country of birth. Descriptive analyses with chi2 tests and binary logistic regression analyses were performed.

**Results:**

Adolescents with perceived parental alcohol problems had higher odds of reporting psychosomatic complaints compared with adolescents without perceived parental drinking problems, even when adjusting for sociodemographic characteristics. Girls, grade 11 students, adolescent with at least one parent born in Sweden, and those without university-educated parents were more likely to report parental alcohol problems.

**Conclusions:**

The findings highlight adolescents with perceived parental alcohol problems need support. The school, being an arena where adolescents spend much of their time, may play a vital role in this regard.

## Highlights

• Knowledge is lacking on the health of youth reporting parental problem drinking.
• Data from a Swedish nationally representative sample of 9,032 teens were used.
• Perceived parental alcohol problems were associated with psychosomatic complaints.
• Attention and support to this group of adolescents is important.

## 1 Introduction

Adverse childhood experiences (ACEs) have a profound impact on individuals’ lives both in the short and in the long run (Boullier and Blair, 2018, Petruccelli et al., 2019). One particular type of adversity relates to alcohol problems in the family of origin (Felitti et al., 1998). Children of problem-drinking parents have increased risks of short- and long-term adverse outcomes in terms of, e.g., higher levels of drinking and more problematic drinking themselves (Haugland et al., 2013, Karlsson et al., 2016, Olssonet al., 2019, Olsson et al., 2021, Pisinger et al., 2017, Thor et al., 2022), higher levels of self-reported health problems (Pisinger et al., 2016, Wahlström et al., 2023a), increased risks of mental and behavioural disorders (Johnson and Leff, 1999, Landberg et al., 2019, Raitasalo et al., 2019), as well as poorer educational outcomes (Berg et al., 2016, Pisinger et al., 2023).

Living with a parent with alcohol problems can be regarded as a chronic stressor with potentially negative implications for health. Children of problem-drinking parents might have a challenging family situation with a poor sense of belonging, poor control over the situation, and lack of parental support (Windle, 1996, Lee and Williams, 2013). Indeed, adolescents with perceived parental alcohol problems have reported poorer parent–child relationships compared with adolescents without such problems (Pisinger et al., 2016, Wahlström et al., 2023b). The family situation may also involve feelings of shame, stigma and guilt, which could lead to social isolation (Hutchinson et al., 2014, Tamutienė and Jogaitė, 2019). Furthermore, adolescents with perceived parental alcohol problems have increased risks of being subjected to bullying (Ramstedt et al., 2022).

Psychosomatic complaints have been shown to correlate with perceived stress in adolescents, and can hence be regarded as an indicator of stress-related ill-health in this age group (Corell et al., 2022). To date, the association between parental alcohol problems and adolescents’ self-reported psychosomatic complaints has been scarcely investigated, although there are some recent studies based on Swedish data which report links between parental alcohol problems and children’s psychosomatic complaints (Ramstedt et al., 2023, Wahlström et al., 2023b, Ramstedt et al., 2022, Wahlström et al., 2023a). Overall, however, there is limited knowledge and a relative lack of empirical evidence on this topic.

The aim of the current study was to examine perceived parental alcohol problems and the links with psychosomatic complaints among Swedish adolescents aged 15–18 years. The research questions are:

1) How common are perceived parental alcohol problems among Swedish adolescents, and does the reporting of such problems differ by sociodemographic characteristics?
2) Are perceived parental alcohol problems associated with a higher likelihood of reporting psychosomatic complaints, whilst adjusting for sociodemographic characteristics?

## 2 Material and methods

The data was drawn from the 2021 national school survey performed by the Swedish Council for Information on Alcohol and Other Drugs (CAN). The survey has been conducted yearly since 1971 in grade 9 (ages 15–16 years) and since 2004 also in grade 11 (ages 17–18 years) (CAN, 2021). The aim of the survey is to collect information about the consumption of alcohol, narcotics, tobacco, and doping use as well as about gambling experiences among adolescents. Since 2019, there has been a transition from pen and paper to a digital form of data collection, and in 2021, a complete digital data collection was performed for the first time.

Every year, CAN draws a random sample of 350 schools in each grade. In 2021, the number of students in the classes participating in the survey was 6,609 in grade 9 and 5,427 in grade 11. The number of absent students reported by teachers was 1,253 in grade 9 (19 %) and 1,041 in grade 11 (19 %). The number of students who refrained from participating in the survey was 11 in grade 9 (0.2 %), and 9 in grade 11 (0.2 %). The number of students sorted out from the survey due to inconsistent, exaggerated, or unrealistic answers was 91 in grade 9 (1.4 %), and 28 in grade 11 (0.5 %) (CAN, 2021). Accordingly, our data included 9,603 students (n = 5,254 in grade 9; n = 4,349 in grade 11). Out of these, 571 cases with missing information were excluded, rendering an analytical sample of 9,032 students (n = 4,892 in grade 9 and n = 4,140 in grade 11).

### 2.1 Ethics

CAN instructed all schools to perform the questionnaire in the classroom under examination-like conditions. Students used a one-time password to log in to the survey. The password was linked to a class but not to an individual, and the students did not include their names or any other personal information in the survey. The students received information that participation was voluntary. Parental consent was not obtained since according to the Swedish Ethical Review Act (SFS, 2003:460), this is not required for adolescents aged 15 or older if they realise what the research means for them.

### 2.2 Measures

Psychosomatic complaints were captured by the question: “During the past six months, how often have you…” and the items a) “had a headache”; b) “had a stomach ache”; c) “felt depressed or down”; d) “had difficulties to fall asleep”; and e) “slept poorly at night”. The response categories were “Nearly every day”; “Several times a week”; “Once a week”; “Some time/a few times a month”; and “Rarely or never”. In line with commonly used measures of psychosomatic complaints such as the Health Behaviour in School-aged Children (HBSC) symptom checklist (HBSC-SCL) (e.g., Public Health Agency of Sweden, 2019, Cosma et al., 2020) and the Psychosomatic Problems (PSP) scale (Corell et al., 2022, Hagquist, 2009) the variable covers different types of complaints (i.e., psychological as well as somatic ones). The five items were weakly to moderately correlated (Pearson’s r ranging between 0.34 and 0.50) except for the two items about sleep which were highly correlated (r = 0.75). Internal consistency was acceptable (Cronbach’s α = 0.71). Each item was dichotomised to distinguish between students who reported the complaint more than weekly (i.e. nearly every day or several times a week) vs. less often. In addition, we constructed a composite measure that distinguished between students who reported two or more psychosomatic complaints more than weekly vs. others. Dichotomization makes the interpretation of the results easier to understand, and odds ratios are a meaningful measure of association (Farrington & Loeber, 2000).

Perceived parental alcohol problems were measured by the Children of Alcoholics Screening Test (CAST-6) scale (Hodgins & Shimp, 1995). The CAST-6 scale includes six yes/no statements instead of the original scale of 30 items. This short version has proven to be as effective as the original version (Hodgins & Shimp, 1995). These six questions cover four areas: perceptions of parental alcohol problems; attempts to control parental drinking; perceptions of marital discord; and efforts to escape adverse consequences. Internal consistency was high (Cronbach’s α = 0.87). We created a binary variable distinguishing between students who answered affirmatively to at least three of the six statements vs. those who answered affirmatively to less than three of the statements (Hodgins and Shimp, 1995, Ramstedt et al., 2022).

Sociodemographic background characteristics included adolescents’ gender (male; female; and other), grade (9 and 11), parental education (at least one parent with a university/college education level; no parent has a university/college-level education; and do not know), and parental country of birth (at least one parent born in Sweden; two parents born abroad; and do not know).

### 2.3 Statistical method

The prevalence of perceived parental alcohol problems by sociodemographic characteristics was evaluated by chi-square tests. The associations between perceived parental alcohol problems and psychosomatic complaints were examined by means of cross-tabulations and chi-square tests as well as by binary logistic regression analyses, adjusting for sociodemographic background characteristics. To take the clustered nature of the data into account, with students nested in classes, robust standard errors were estimated in the logistic regression analyses. The analyses were performed with Stata, version 17 (StataCorp, 2021).

## 3 Results

Table 1 shows descriptive statistics of the study sample. Overall, 34.6 % of adolescents experienced two or more psychosomatic symptoms weekly. Regarding the CAST-6 questions about parental alcohol problems, 12.9 % of adolescents responded affirmatively to three or more of these. For the specific items, the proportions who answered affirmatively varied between 20.6 % (“Parent drank too much”) and 7.7 % (“Wanted to hide/pour parent’s alcohol bottle”).

**Table 1:** Descriptives of the study sample. (n = 9,032).

	n	%
**Psychosomatic complaints (more than weekly)**		
Headache	1,800	19.9
Stomach ache	1,385	15.3
Depressed and down	2,649	29.3
Difficulty in falling asleep	2,731	30.2
Slept poorly at night	2,433	26.9
Psychosomatic complaints (≥2)	3,129	34.6
**Perceived parental alcohol problems (CAST-6)**		
Parent drank too much	1,862	20.6
Asked a parent to stop drinking	1,127	12.5
Quarrelled with parent while drinking	1,098	12.2
Heard parents arguing while one is drunk	1,138	12.6
Wanted to hide/pour parent’s alcohol bottle	693	7.7
Wish that a parent stopped drinking	1,200	13.3
CAST-6 (cutoff ≥ 3)	1,169	12.9
**Sociodemographic characteristics**		
Gender		
Boys	4,387	48.6
Girls	4,509	49.9
Other	136	1.5
Grade		
9	4,892	54.2
11	4,140	45.8
Parents’ country of birth		
At least one in Sweden	7,401	81.9
Two abroad	1,527	16.9
Do not know	104	1.2
Parents’ university/college education		
At least one parent	6,515	72.1
No one	1,103	12.2
Do not know	1,414	15.7

Table 2 displays crosstabulations with chi-square tests of perceived parental alcohol problems (CAST-6) by sociodemographic characteristics. Perceived parental alcohol problems were more common among girls and among those with another gender identity compared with boys (p < 0.001), among students in grade 11 compared with those in grade 9 (p < 0.01), among adolescents with at least one parent born in Sweden compared with those with two parents born abroad (p < 0.001), and among those with no university-educated parent and those who responded “don’t know” to the questions about parental education, compared with those who had at least one parent with a university education (p < 0.001).

**Table 2:** Crosstabs between CAST-6 and socio-demographic characteristics (n = 9,032).

	Parental alcohol drinking problems CAST-6 sum at cut-off 3				
	Less than three		At least three		χ^2^
	N	%	n	%	
Gender					
Boys	3,999	91.2	388	8.8	
Girls	3,748	83.1	761	16.9	
Other	116	85.3	20	14.7	127.7***
Grade					
9	4,302	87.9	590	12.1	
11	3,561	86.0	579	14.0	7.4**
Parent’s country of birth					
At least one in Sweden	6,376	86.2	1,025	13.9	
Two abroad	1,394	91.3	133	8.7	
Don’t know	93	89.4	11	10.6	30.2***
Parents’ university/college education educationeducation					
At least one parent	5,745	88.2	770	11.8	
No one	932	84.5	171	15.5	
Do not know	1,186	83.9	228	16.1	26.4***

**p < 0.01, ***p < 0.001.

Adolescents’ psychosomatic complaints (≥2 more than weekly) by perceived parental alcohol problems (CAST-6) are presented in Table 3. Psychosomatic complaints were more common among adolescents who had responded affirmatively to the CAST-6 items, compared with those who had not. This was true for each individual CAST-6 item as well as for the composite CAST-6 measure. All differences between groups were statistically significant (p < 0.001).

**Table 3:** Adolescents’ psychosomatic complaints (≥2 more than weekly) by perceived parental alcohol problems (CAST-6). Per cent and χ^2^ tests of statistically significant differences between groups. (n = 9,032).

	Psychosomatic complaints (≥2 more than weekly) ( %)		χ^2^
	No	Yes	
Parent drank too much			
No	68.2	31.8	
Yes	54.3	45.7	126.7***
Asked a parent to stop drinking			
No	67.4	32.6	
Yes	50.8	49.3	121.3***
Quarrelled with parent while drinking			
No	67.8	32.2	
Yes	47.5	52.5	175.2***
Heard parents arguing while one is drunk			
No	67.5	32.5	
Yes	50.3	49.7	131.0***
Wanted to hide/pour parent’s alcohol bottle			
No	67.0	33.1	
Yes	46.2	53.8	122.0***
Wish that a parent stopped drinking			
No	67.3	32.7	
Yes	52.5	47.5	101.0***
Perceived parental alcohol problems (CAST-6, cutoff ≥ 3)			
No (ref.)	67.9	32.1	
Yes	48.4	51.6	170.2***

***p < 0.001.

Table 4 presents results from binary logistic regression analyses, with each individual psychosomatic complaint and the composite measure (≥2 complaints more than weekly) regressed on perceived parental alcohol problems, adjusted for sociodemographic characteristics. Perceived parental alcohol problems were positively and significantly associated with each psychosomatic complaint (headache OR 1.67, 95 % CI 1.45–1.92; stomach ache OR 1.67, 95 % CI 1.44–1.93; depressed and down OR 2.08, 95 % CI 1.82–2.38; difficulty in falling asleep OR 1.66, 95 % CI 1.46–1.88, slept poorly at night OR 1.79, 95 % CI 1.58–2.02) as well as with the composite measure of psychosomatic complaints (OR 2.04, 95 % CI 1.80–2.32). Additional analyses with a less restrictive cut-off for CAST 6 (≥2 affirmative answers on individual items) showed slightly weaker but overall similar associations (see Appendix, Table A1). We also performed a set of linear regression analyses of the psychosomatic complaints operationalised as continuous measures (see Appendix, Table A2). In these analyses, each complaint was coded 1–5, with higher values indicating more frequent complaints. In addition, we created a continuous measure adding the values of all five complaints to a measure of psychosomatic complaints ranging 5–25. As seen in Table A2, the results from the linear regression analyses largely reflect those from the binary logistic regression analyses.

**Table 4:** Adolescents’ psychosomatic complaints (more than weekly) regressed on perceived parental alcohol problems (CAST-6, cutoff ≥ 3). Odds ratios (OR) and 95 % confidence intervals (95 % CI) from binary logistic regression models with robust standard errors. (n = 9,032).

	Headache	Stomach ache	Depressed and down	Difficulty in falling asleep	Slept poorly at night	Psychosomatic complaints (≥2)
	OR	OR	OR	OR	OR	OR
	(95 % CI)	(95 % CI)	(95 % CI)	(95 % CI)	(95 % CI)	(95 % CI)
Perceived parental alcohol problems (CAST-6, cutoff ≥ 3)						
No (ref.)	1.00	1.00	1.00	1.00	1.00	1.00
Yes	1.67***	1.67***	2.08***	1.66***	1.79***	2.04***
	(1.45–1.92)	(1.44–1.93)	(1.82–2.38)	(1.46–1.88)	(1.58–2.02)	(1.80–2.32)
Gender						
Boys (ref.)	1.00	1.00	1.00	1.00	1.00	1.00
Girls	3.64***	4.41***	3.33***	1.53***	1.46***	2.39***
	(3.22–4.13)	(3.80–5.12)	(2.98–3.71)	(1.39–1.70)	(1.32–1.61)	(2.17–2.63)
Other	3.86***	5.00***	3.40***	2.00***	1.94***	2.63***
	(2.64–5.64)	(3.28–7.60)	(2.36–4.90)	(1.43–2.81)	(1.35–2.80)	(1.83–3.80)
Grade						
9 (ref.)	1.00	1.00	1.00	1.00	1.00	1.00
11	0.81***	0.89	0.86*	0.93	0.93	0.88*
	(0.73–0.91)	(0.78–1.02)	(0.77–0.96)	(0.84–1.03)	(0.84–1.03)	(0.79–0.98)
Parents’ country of birth						
At least one in Sweden (ref).	1.00	1.00	1.00	1.00	1.00	1.00
Two abroad	1.19*	1.10	0.99	1.15*	1.64***	1.29***
	(1.02–1.38)	(0.93–1.30)	(0.86–1.14)	(1.01–1.31)	(1.45–1.86)	(1.14–1.47)
Do not know	1.90*	1.56	2.08***	1.72**	2.07**	2.11**
	(1.16–3.10)	(0.88–2.75)	(1.38–3.12)	(1.15–2.56)	(1.37–3.13)	(1.43–3.11)
Parents’ university/college education						
At least one parent (ref.)	1.00	1.00	1.00	1.00	1.00	1.00
No one	1.01	0.99	0.88	1.08	1.08	1.01
	(0.86–1.20)	(0.83–1.18)	(0.76–1.02)	(0.94–1.25)	(0.94–1.24)	(0.88–1.16)
Do not know	0.97	0.92	1.02	1.17*	1.15*	1.04
	(0.83–1.13)	(0.78–1.08)	(0.90–1.15)	(1.03–1.33)	(1.01–1.31)	(0.92–1.17)

*p < 0.05, **p < 0.01, ***p < 0.001.

Subsequently, we included the interactions terms between perceived parental alcohol problems and gender and grade, one at a time, in the fully adjusted models. Through Wald tests, we found that gender moderated the associations between perceived parental alcohol problems and having a headache (p < 0.01) and feeling depressed and down (p < 0.05), while grade moderated the associations between parental alcohol problems and difficulties falling asleep (p < 0.05) and the composite measure of psychosomatic complaints (p < 0.01). Further analyses stratified by gender and grade revealed that the associations in question were stronger for boys compared with girls, and for students in grade 9 compared with students in grade 11, respectively (see Appendix, Tables A3–A7).

## 4 Discussion and conclusion

This study aimed to examine perceived parental alcohol problems and the links with psychosomatic complaints among Swedish adolescents aged 15–18 years. The results demonstrated that adolescents with perceived parental alcohol problems had about twice as high odds of reporting psychosomatic complaints compared with adolescents without perceived parental alcohol problems, even when adjusting for sociodemographic background characteristics. This finding reflects those of recent studies based on other Swedish data materials using different measures of parental alcohol problems (Ramstedt et al., 2022, Ramstedt et al., 2023, Wahlström et al., 2023a, Wahlström et al., 2023b). While prior studies on CAST-6 and adolescent psychosomatic complaints were based on data collected among grade 9 students (aged 15–16) (Ramstedt et al., 2022, Ramstedt et al., 2023), the present study utilised data from students in both grades 9 and 11, thus expanding the scope of generalisability.

One key mechanism in the association between perceived parental alcohol problems and psychosomatic complaints is likely to be stress. Not only may parental alcohol problems be stressful in themselves, but they may also imply that the children have fewer resources that can help to cope with stress. Indeed, children who experience parental alcohol problems are at a risk of having poor family relationships, poor control over the situation in the life, and lack of parental support, possibly leading to a shortage of protective buffering factors and poor development of coping and resilience skills against stress in life (Windle, 1996, Lee and Williams, 2013). Furthermore, children may feel shame, stigma, and guilt because of their parents’ drinking problems (Hutchinson et al., 2014, Tamutienė and Jogaitė, 2019). They may also be at an increased risk of bullying victimisation (Ramstedt et al., 2022). A child might try to avoid inviting friends home to reduce the risk for embarrassing situations and prevent peers from learning about the parents’ alcohol problems, which could potentially lead to social isolation and/or exclusion. Parental alcohol problems can also be assumed to fall under the broader concept of adverse childhood experiences (ACEs) (Felitti et al., 1998). ACEs have consistently been linked to later adverse health outcomes and the proposed pathways highlight that the exposure to stressful events can lead to several negative biological responses followed by social, emotional, and cognitive impairment as well as the adoption of different health-risk behaviours (Boullier and Blair, 2018, Petruccelli et al., 2019). These kinds of consequences may, in turn, be manifested as psychosomatic complaints in the affected adolescents.

The current study also found that perceived parental alcohol problems was more commonly reported by girls, non-binary adolescents, students in grade 11, those with at least one parent born in Sweden, and respondents without university-educated parents. While previous research has found differences in perceived parental alcohol problems by gender (Haugland and Elgan, 2021, Pisinger et al., 2016, Ramstedt et al., 2022), the explanations behind this are not clear. Possible mechanisms could include that girls are more likely to disclose family-related behaviour or exposure to harmful experiences (Lev-Wiesel and First, 2018, van der Ploeg et al., 2022) and are more sensitive to parental drinking problems (Homel & Warren, 2019). Similarly, older adolescents could also be more likely to recognise and reveal parental drinking problems and the conflicts associated with it compared with younger children. In the case of adolescents with parents born abroad, they are a diverse group with differing background and cultural traditions. Nevertheless, alcohol abuse in Sweden tend to be less prevalent among migrants compared to the native population (Harris et al., 2019), which could explain our finding. Regarding parental education, prior research in the Swedish context has demonstrated that while individuals with a higher education drink more alcohol in total, they tend to drink less intensely compared with individuals with a lower education (Heckley et al., 2017). Higher educational attainment is also associated with a lower risk of alcohol use disorder in Sweden (Calling et al., 2019). Thus, adolescents with parents who have not studied at university may indeed experience more problems related to their parents’ drinking.

Additionally, we found that the association between perceived parental alcohol problems and (some) psychosomatic complaints was stronger for boys and for students in grade 9. Since boys and younger students were less likely to report perceived parental alcohol problems, it is possible that more severe cases of parental drinking problems were captured for these adolescents. Some other possible explanations include that boys do not experience the same level of stress from, e.g., school, as girls and might therefore be more susceptible to other forms of stressors (Östberg et al., 2015). Younger students tend to be more dependent on their parents and may thus be more strongly affected by parental problem drinking compared with older adolescents who are probably more independent and have stronger bonds with peers.

To extend current knowledge, longitudinal, prospective studies that are based on children’s and adolescents’ reports in childhood, and that follow the offspring until adulthood would be beneficial. Qualitative research might also improve the contextual understanding of the mechanisms between parental alcohol problems and children’s self-reported health. Finally, research should investigate how the association between perceived parental alcohol problems and offspring health can be alleviated. It seems highly relevant to map how social support from, e.g., teachers, other school staff, and leaders in extra-curricular activities may buffer against problematic conditions in the family, and to propose how such knowledge may be translated into policy and practice.

### 4.1 Strengths and limitations

One strength of the current study is that the measure of parental alcohol problems was based on adolescents’ survey responses. It is not uncommon to use data on treatment for alcohol-related disorders to measure parental alcohol problems. Since a majority of individuals with mild to moderate alcohol dependence do not seem to seek help and treatment (Wallhed Finn et al., 2014), such measures are however biased to the most severe (clinical) cases and findings may therefore have limited generalisability to the population at large. At the same time, it should be acknowledged that self-reported data may be conflated by bias. For instance, the present study’s finding that girls (and participants who marked the “other” gender category) were more likely than boys to report perceived parental alcohol problems may possibly be due to gender differences in perceptions and/or reporting rather than in actual situations (for a discussion, see Wahlström et al., 2023b).

The large-scale nationally representative data, with a high response rate at the student level, can also be regarded as a strength. Notwithstanding, it should be acknowledged that the non-response was most likely systematic. First, it is reasonable to assume that students with a problematic situation at home and/or psychosomatic complaints were probably more likely to be absent on the day of the survey, implying a systematic bias in the non-response. Second, with regards to the students in grade 11 in our sample, there may be an additional bias as upper secondary school is not mandatory in Sweden. Although the majority of adolescents enrol in upper secondary school, it is likely that students with problematic familial alcohol use are more inclined to not continue to or to drop out from upper secondary school (Pisinger et al., 2023). Relatedly, this group of adolescents probably also have more psychosomatic complaints. However, these possible biases are more likely to imply underestimations of the associations, rather than the other way around.

Due to the cross-sectional study design, we were not able to establish a temporal sequence or draw conclusions about causality with support in the data. Yet, it seems implausible that adolescents’ poor psychosomatic health causes parental alcohol problems. Nonetheless, we cannot rule out the possibility that there are omitted variables that may impact the reported associations, for instance, parents’ mental health problems or (other aspects of) family psychosocial adversity.

Another limitation is that CAST-6 is a life time measurement scale with no referral to a specific time frame, whereas the questions about psychosomatic complaints refer to last six months. Hence, mapping a life time measure scale against six months confined psychosomatic complaints can have temporal validity issues.

Furthermore, it should be acknowledged that the data used in the present study was collected during the covid-19 pandemic, which may have affected the generalisability of the results. Even though no significant change in the alcohol consumption among the general adult population in Sweden during the pandemic has been detected (Guttormsson, 2022, Trolldal, 2022), it is possible that young people’s lives were affected by covid-19 in various ways. For instance, due to the social distance recommendations (and to some extent, also distance learning at school), people in general including adolescents were more bound to their homes during this time than usual. It is possible to assume that social support from friends but also from teachers and other school staff was less accessible. Hence the potentially stress-buffering role of social support on the association between parental drinking and adolescent psychosomatic complaints may have been less prominent than at other times. Notwithstanding, the findings regarding both the prevalence of perceived parental alcohol problems and the associations with psychosomatic complaints in the present study are very similar those reported by Ramstedt et al., 2022, Ramstedt et al., 2023 which were based on survey data collected prior to the pandemic.

### 4.2 Conclusions

The findings of the present study highlight that adolescents with perceived parental alcohol problems have an increased risk of reporting psychosomatic complaints, and hence this group should be given adequate attention and support. The school health services could play an important role in identifying students who are living with problem-drinking parents and school-based interventions may be effective in mitigating against the adverse effects of parental drinking (cf. Låftman et al., 2021). Other ways for policy makers to address this issue is to target harmful parental drinking in general and to provide children in need with sufficient opportunities to seek and receive help.

## CRediT authorship contribution statement

**Numan Raza Syed:** Conceptualization, Methodology, Formal analysis, Writing – original draft. **Joakim Wahlström:** Formal analysis, Writing – review & editing. **Sara Brolin Låftman:** Conceptualization, Methodology, Writing – review & editing, Supervision. **Johan Svensson:** Conceptualization, Methodology, Writing – review & editing, Supervision.

## Declaration of Competing Interest

The authors declare that they have no known competing financial interests or personal relationships that could have appeared to influence the work reported in this paper.

## Data Availability

The data are not publicly available. Access to the data can be applied for at the Swedish Council for Information on Alcohol and Other Drugs (CAN), under the condition that the Swedish Ethical Review Authority has granted permission.

## Appendix A Supplementary material

Supplementary data 1 Appendix Tables A1–A7.
