# Supplementary material for: Perceived parental alcohol problems and psychosomatic complaints among adolescents in Sweden
Source: Addict Behav Rep. 2023 Apr 24;17:100491. doi: 10.1016/j.abrep.2023.100491 (PMC10163609; doi:10.1016/j.abrep.2023.100491)
Supplement: Supplementary data 1 — Appendix Tables A1–A7. [file mmc1.docx]

# Appendix

Table A1. Adolescents’ psychosomatic complaints (more than weekly) regressed on perceived parental alcohol problems (CAST-6, cutoff ≥2). Odds ratios (OR) and 95% confidence intervals (95% CI) from binary logistic regression models with robust standard errors. (n= 9,032)

|  | Headache | Stomach ache | Depressed and down | Difficulty in falling asleep | | Slept poorly at night | | Psychosomatic complaints (≥2) |
| --- | --- | --- | --- | --- | --- | --- | --- | --- |
|  | OR | OR | OR | OR | OR | | OR | |
|  | (95% CI) | (95% CI) | (95% CI) | (95% CI) | (95% CI) | | (95% CI) | |
| Perceived parental alcohol problems (CAST-6, cutoff ≥2) |  |  |  |  |  | |  | |
| No (ref.) | 1.00 | 1.00 | 1.00 | 1.00 | 1.00 | | 1.00 | |
| Yes (18.5%) | 1.55*** | 1.53*** | 1.95*** | 1.56*** | 1.64*** | | 1.83*** | |
|  | (1.36-1.77) | (1.34-1.76) | (1.73-2.19) | (1.39-1.75) | (1.47-1.84) | | (1.63-2.05) | |
| Gender |  |  |  |  |  | |  | |
| Boys (ref.) | 1.00 | 1.00 | 1.00 | 1.00 | 1.00 | | 1.00 | |
| Girls | 3.63*** | 4.40*** | 3.30*** | 1.53*** | 1.45*** | | 2.38*** | |
|  | (3.20-4.11) | (3.79-5.11) | (2.96-3.67) | (1.38-1.69) | (1.31-1.61) | | (2.16-2.62) | |
| Other | 3.84*** | 4.97*** | 3.37*** | 1.99*** | 1.93*** | | 2.61*** | |
|  | (2.64-5.60) | (3.26-7.57) | (2.34-4.84) | (1.42-2.79) | (1.34-2.77) | | (1.81-3.77) | |
| Grade |  |  |  |  |  | |  | |
| 9 (ref.) | 1.00 | 1.00 | 1.00 | 1.00 | 1.00 | | 1.00 | |
| 11 | 0.81*** | 0.89 | 0.86* | 0.93 | 0.93 | | 0.88* | |
|  | (0.73-0.91) | (0.78-1.02) | (0.77-0.97) | (0.84-1.04) | (0.84-1.03) | | (0.80-0.98) | |
| Parents’ country of birth |  |  |  |  |  | |  | |
| At least one in Sweden (ref). | 1.00 | 1.00 | 1.00 | 1.00 | 1.00 | | 1.00 | |
| Two abroad | 1.20* | 1.11 | 1.01 | 1.16* | 1.66*** | | 1.31*** | |
|  | (1.03-1.40) | (0.94-1.31) | (0.88-1.16) | (1.02-1.32) | (1.46-1.88) | | (1.15-1.49) | |
| Do not know | 1.92* | 1.57 | 2.12*** | 1.74** | 2.10*** | | 2.14*** | |
|  | (1.17-3.15) | (0.89-2.78) | (1.41-3.19) | (1.17-2.59) | (1.39-3.17) | | (1.45-3.16) | |
| Parents’ university/ college education |  |  |  |  |  | |  | |
| At least one parent (ref.) | 1.00 | 1.00 | 1.00 | 1.00 | 1.00 | | 1.00 | |
| No one | 1.02 | 0.98 | 0.86 | 1.07 | 1.07 | | 1.00 | |
|  | (0.85-1.19) | (0.82-1.17) | (0.74-1.00) | (0.93-1.24) | (0.93-1.23) | | (0.87-1.15) | |
| Do not know | 0.96 | 0.91 | 1.01 | 1.17* | 1.14* | | 1.03 | |
|  | (0.83-1.12) | (0.78-1.08) | (0.89-1.15) | (1.03-1.32) | (1.01-1.30) | | (0.91-1.17) | |

*p<0.05, **p<0.01, ***p<0.001

Table A2. Adolescents’ psychosomatic complaints (more than weekly) regressed on perceived parental alcohol problems (CAST-6, cutoff ≥3). Coefficients with 95% confidence interval (95% CI) from linear regression models with robust standard errors. (n= 9,032).

|  | Headache | Stomach ache | Depressed and down | Difficulty in falling asleep | | Slept poorly at night | | Psychosomatic complaints (≥2) |
| --- | --- | --- | --- | --- | --- | --- | --- | --- |
|  | *b* | *b* | *b* | *b* | *b* | | *b* | |
|  | (95% CI) | (95% CI) | (95% CI) | (95% CI) | (95% CI) | | (95% CI) | |
| Perceived parental alcohol problems (CAST-6, cutoff ≥3) |  |  |  |  |  | |  | |
| No (ref.) | 1.00 | 1.00 | 1.00 | 1.00 | 1.00 | | 1.00 | |
| Yes (18.5%) | 0.27*** | 0.28*** | 0.53*** | 0.39*** | 0.42*** | | 1.89*** | |
|  | (0.20, 0.34) | (0.21, 0.35) | (0.45, 0.61) | (0.30, 0.47) | (0.34, 0.50) | | (1.60, 2.17) | |
| Gender |  |  |  |  |  | |  | |
| Boys (ref.) | 1.00 | 1.00 | 1.00 | 1.00 | 1.00 | | 1.00 | |
| Girls | 0.72*** | 0.77*** | 0.84*** | 0.29*** | 0.28*** | | 2.90*** | |
|  | (0.67, 0.77) | (0.72, 0.82) | (0.78, 0.90) | (0.23, 0.35) | (0.22, 0.34) | | (2.69, 3.11) | |
| Other | 0.68*** | 0.66*** | 0.77*** | 0.48*** | 0.38** | | 2.97*** | |
|  | (0.44, 0.91) | (0.43, 0.89) | (0.51, 1.02) | (0.23, 0.74) | (0.13, 0.63) | | (1.96, 3.97) | |
| Grade |  |  |  |  |  | |  | |
| 9 (ref.) | 1.00 | 1.00 | 1.00 | 1.00 | 1.00 | | 1.00 | |
| 11 | -0.08** | -0.07** | -0.02 | -0.02 | 0.01 | | -0.18 | |
|  | (-0.13,  -0.03) | (-0.12,  -0.03) | (-0.08,  -0.04) | (-0.09, 0.04) | (-0.05,  0.07) | | (-0.39, 0.03) | |
| Parents’ country of birth |  |  |  |  |  | |  | |
| At least one in Sweden (ref). | 1.00 | 1.00 | 1.00 | 1.00 | 1.00 | | 1.00 | |
| Two abroad | 0.07** | 0.04 | -0.12** | 0.06 | 0.28*** | | 0.33* | |
|  | (0.01, 0.14) | (-0.02, 0.10) | (-0.19, -0.04) | (-0.02, 0.14) | (0.20, 0.35) | | (0.06, 0.61) | |
| Do not know | 0.42** | 0.28* | 0.30* | 0.31* | 0.47** | | 1.78*** | |
|  | (0.18, 0.66) | (0.07, 0.49) | (0.03, 0.56) | (0.03, 0.59) | (0.19, 0.76) | | (0.81, 2.75) | |
| Parents’ university/ college education |  |  |  |  |  | |  | |
| At least one parent (ref.) | 1.00 | 1.00 | 1.00 | 1.00 | 1.00 | | 1.00 | |
| No one | 0.03 | -0.01 | -0.11** | 0.04 | 0.02 | | -0.02 | |
|  | (-0.05, 0.10) | (-0.07, 0.06) | (-0.19, -0.04) | (-0.04, 0.13) | (-0.06, 0.11) | | (-0.30, 0.25) | |
| Do not know | -0.03 | -0.05 | -0.06 | 0.09* | 0.08* | | 0.03 | |
|  | (-0.10, 0.04) | (-0.11, 0.01) | (-0.13, 0.01) | (0.01, 0.17) | (0.01, 0.15) | | (-0.21, 0.27) | |

*p<0.05, **p<0.01, ***p<0.001

Table A3. Adolescents’ psychosomatic complaints (more than weekly) regressed on perceived parental alcohol problems (CAST-6, cutoff ≥3). Odds ratios (OR) and 95% confidence intervals (95% CI) from binary logistic regression models with robust standard errors. Boys only (n= 4,387)

|  | Headache | Stomach ache | Depressed and down | Difficulty in falling asleep | | Slept poorly at night | | Psychosomatic complaints (≥2) |
| --- | --- | --- | --- | --- | --- | --- | --- | --- |
|  | OR | OR | OR | OR | OR | | OR | |
|  | (95% CI) | (95% CI) | (95% CI) | (95% CI) | (95% CI) | | (95% CI) | |
| Perceived parental alcohol problems (CAST-6, cutoff ≥3) |  |  |  |  |  | |  | |
| No (ref.) | 1.00 | 1.00 | 1.00 | 1.00 | 1.00 | | 1.00 | |
| Yes | 2.42*** | 1.69** | 2.57*** | 1.81*** | 1.92*** | | 2.25*** | |
|  | (1.85-3.17) | (1.14-2.50) | (2.05-3.21) | (1.45-2.26) | (1.54-2.40) | | (1.83-2.77) | |
| Grade |  |  |  |  |  | |  | |
| 9 (ref.) | 1.00 | 1.00 | 1.00 | 1.00 | 1.00 | | 1.00 | |
| 11 | 0.78* | 0.74* | 0.94 | 1.04 | 0.94 | | 0.95 | |
|  | (0.63-0.96) | (0.58-0.95) | (0.79-1.11) | (0.88-1.22) | (0.80-1.10) | | (0.81-1.11) | |
| Parents’ country of birth |  |  |  |  |  | |  | |
| At least one in Sweden (ref). | 1.00 | 1.00 | 1.00 | 1.00 | 1.00 | | 1.00 | |
| Two abroad | 1.16 | 0.86 | 0.91 | 0.99 | 1.57*** | | 1.17 | |
|  | (0.90-1.51) | (0.61-1.22) | (0.73-1.13) | (0.82-1.19) | (1.31-1.88) | | (0.97-1.40) | |
| Do not know | 2.89*** | 2.25* | 1.85* | 1.64* | 2.16** | | 2.25** | |
|  | (1.65-5.07) | (1.14-4.44) | (1.12-3.05) | (1.02-2.62) | (1.31-3.57) | | (1.41-3.60) | |
| Parents’ university/ college education |  |  |  |  |  | |  | |
| At least one parent (ref.) | 1.00 | 1.00 | 1.00 | 1.00 | 1.00 | | 1.00 | |
| No one | 1.16 | 1.32 | 0.95 | 1.08 | 1.05 | | 1.10 | |
|  | (0.85-1.58) | (0.93-1.89) | (0.74-1.23) | (0.87-1.34) | (0.85-1.31) | | (0.88-1.37) | |
| Do not know | 0.84 | 0.98 | 1.07 | 1.14 | 1.21* | | 1.11 | |
|  | (0.65-1.10) | (0.71-1.37) | (0.86-1.33) | (0.94-1.38) | (1.01-1.46) | | (0.92-1.33) | |

*p<0.05, **p<0.01, ***p<0.001

Table A4. Adolescents’ psychosomatic complaints (more than weekly) regressed on perceived parental alcohol problems (CAST-6, cutoff ≥3). Odds ratios (OR) and 95% confidence intervals (95% CI) from binary logistic regression models with robust standard errors. Girls only (n= 4,509)

|  | Headache | Stomach ache | Depressed and down | Difficulty in falling asleep | | Slept poorly at night | | Psychosomatic complaints (≥2) |
| --- | --- | --- | --- | --- | --- | --- | --- | --- |
|  | OR | OR | OR | OR | OR | | OR | |
|  | (95% CI) | (95% CI) | (95% CI) | (95% CI) | (95% CI) | | (95% CI) | |
| Perceived parental alcohol problems (CAST-6, cutoff ≥3) |  |  |  |  |  | |  | |
| No (ref.) | 1.00 | 1.00 | 1.00 | 1.00 | 1.00 | | 1.00 | |
| Yes | 1.46*** | 1.62*** | 1.88*** | 1.55*** | 1.76*** | | 1.92*** | |
|  | (1.22-1.73) | (1.38-1.90) | (1.60-2.21) | (1.32-1.82) | (1.51-2.05) | | (1.64-2.26) | |
| Grade |  |  |  |  |  | |  | |
| 9 (ref.) | 1.00 | 1.00 | 1.00 | 1.00 | 1.00 | | 1.00 | |
| 11 | 0.83* | 0.95 | 0.82** | 0.85* | 0.92 | | 0.85* | |
|  | (0.72-0.96) | (0.81-1.12) | (0.71-0.94) | (0.75-0.97) | (0.80-1.05) | | (0.74-0.96) | |
| Parents’ country of birth |  |  |  |  |  | |  | |
| At least one in Sweden (ref). | 1.00 | 1.00 | 1.00 | 1.00 | 1.00 | | 1.00 | |
| Two abroad | 1.17 | 1.18 | 1.03 | 1.29** | 1.71*** | | 1.39*** | |
|  | (0.97-1.41) | (0.97-1.44) | (0.87-1.22) | (1.09-1.54) | (1.45-2.01) | | (1.17-1.65) | |
| Do not know | 0.97 | 0.87 | 4.80** | 2.51* | 2.82** | | 2.48* | |
|  | (0.40-2.33) | (0.32-2.37) | (1.94-11.86) | (1.13-5.59) | (1.30-6.11) | | (1.10-5.60) | |
| Parents’ university/ college education |  |  |  |  |  | |  | |
| At least one parent (ref.) | 1.00 | 1.00 | 1.00 | 1.00 | 1.00 | | 1.00 | |
| No one | 0.98 | 0.87 | 0.85 | 1.07 | 1.08 | | 0.95 | |
|  | (0.80-1.20) | (0.71-1.06) | (0.71-1.02) | (0.88-1.29) | (0.89-1.30) | | (0.79-1.14) | |
| Do not know | 1.02 | 0.88 | 0.98 | 1.17 | 1.06 | | 0.99 | |
|  | (0.85-1.23) | (0.73-1.07) | (0.83-1.17) | (0.97-1.41) | (0.88-1.28) | | (0.82-1.18) | |

*p<0.05, **p<0.01, ***p<0.001

Table A5. Adolescents’ psychosomatic complaints (more than weekly) regressed on perceived parental alcohol problems (CAST-6, cutoff ≥3). Odds ratios (OR) and 95% confidence intervals (95% CI) from binary logistic regression models with robust standard errors. Other gender only (n= 136)

|  | Headache | Stomach ache | Depressed and down | Difficulty in falling asleep | | Slept poorly at night | | Psychosomatic complaints (≥2) |
| --- | --- | --- | --- | --- | --- | --- | --- | --- |
|  | OR | OR | OR | OR | OR | | OR | |
|  | (95% CI) | (95% CI) | (95% CI) | (95% CI) | (95% CI) | | (95% CI) | |
| Perceived parental alcohol problems (CAST-6, cutoff ≥3) |  |  |  |  |  | |  | |
| No (ref.) | 1.00 | 1.00 | 1.00 | 1.00 | 1.00 | | 1.00 | |
| Yes | 3.49* | 5.64*** | 3.26* | 5.14** | 0.98 | | 3.28* | |
|  | (1.35-9.04) | (2.25-14.13) | (1.16-9.16) | (1.86-14.24) | (0.36-2.68) | | (1.21-8.84) | |
| Grade |  |  |  |  |  | |  | |
| 9 (ref.) | 1.00 | 1.00 | 1.00 | 1.00 | 1.00 | | 1.00 | |
| 11 | 0.56* | 0.46 | 1.09 | 0.92 | 0.94 | | 0.60 | |
|  | (0.22-1.44) | (0.15-1.39) | (0.46-2.57) | (0.41-2.06) | (0.43-2.04) | | (0.26-1.40) | |
| Parents’ country of birth |  |  |  |  |  | |  | |
| At least one in Sweden (ref). | 1.00 | 1.00 | 1.00 | 1.00 | 1.00 | | 1.00 | |
| Two abroad | 2.93* | 1.58 | 1.35 | 1.65 | 1.91 | | 1.38 | |
|  | (1.14-7.51) | (0.56-4.42) | (0.56-3.27) | (0.62-4.36) | (0.77-4.79) | | (0.55-3.42) | |
| Do not know | 0.82 | 1.14 | 0.19 | 0.43 | 0.23 | | 0.41 | |
|  | (0.14-4.64) | (0.19-6.78) | (0.02-2.08) | (0.06-2.88) | (0.02-2.74) | | (0.08-2.12) | |
| Parents’ university/ college education |  |  |  |  |  | |  | |
| At least one parent (ref.) | 1.00 | 1.00 | 1.00 | 1.00 | 1.00 | | 1.00 | |
| No one | 0.49 | 3.96* | 0.66 | 2.34 | 1.87 | | 1.24 | |
|  | (0.14-1.70) | (1.23-12.77) | (0.21-2.13) | (0.75-7.34) | (0.59-5.92) | | (0.42-3.69) | |
| Do not know | 1.70 | 1.97 | 1.25 | 2.82* | 2.10 | | 1.14 | |
|  | (0.61-4.72) | (0.61-6.37) | (0.52-3.01) | (1.03-7.71) | (0.81-5.44) | | (0.44-2.96) | |

*p<0.05, **p<0.01, ***p<0.001

Table A6. Adolescents’ psychosomatic complaints (more than weekly) regressed on perceived parental alcohol problems (CAST-6, cutoff ≥3). Odds ratios (OR) and 95% confidence intervals (95% CI) from binary logistic regression models with robust standard errors. Grade 9 only (n= 4,892)

|  | Headache | Stomach ache | Depressed and down | Difficulty in falling asleep | | Slept poorly at night | | Psychosomatic complaints (≥2) |
| --- | --- | --- | --- | --- | --- | --- | --- | --- |
|  | OR | OR | OR | OR | OR | | OR | |
|  | (95% CI) | (95% CI) | (95% CI) | (95% CI) | (95% CI) | | (95% CI) | |
| Perceived parental alcohol problems (CAST-6, cutoff ≥3) |  |  |  |  |  | |  | |
| No (ref.) | 1.00 | 1.00 | 1.00 | 1.00 | 1.00 | | 1.00 | |
| Yes | 1.87*** | 1.83*** | 2.27*** | 1.84*** | 1.96*** | | 2.40*** | |
|  | (1.55-2.26) | (1.51-2.23) | (1.87-2.76) | (1.56-2.18) | (1.64-2.34) | | (2.01-2.87) | |
| Gender |  |  |  |  |  | |  | |
| Boys (ref.) | 1.00 | 1.00 | 1.00 | 1.00 | 1.00 | | 1.00 | |
| Girls | 3.55*** | 3.98*** | 3.55*** | 1.67*** | 1.46*** | | 2.50*** | |
|  | (3.00-4.20) | (3.27-4.85) | (3.06-4.12) | (1.45-1.90) | (1.27-1.67) | | (2.19-2.85) | |
| Other | 4.45*** | 5.07*** | 3.39*** | 2.05** | 1.91** | | 3.03*** | |
|  | (2.80-7.05) | (3.09-8.33) | (2.21-5.20) | (1.36-3.08) | (1.23-2.97) | | (1.93-4.74) | |
| Parents’ country of birth |  |  |  |  |  | |  | |
| At least one in Sweden (ref). | 1.00 | 1.00 | 1.00 | 1.00 | 1.00 | | 1.00 | |
| Two abroad | 1.01 | 0.97 | 0.85 | 0.97 | 1.46*** | | 1.10 | |
|  | (0.82-1.23) | (0.79-1.21) | (0.70-1.02) | (0.81-1.15) | (1.23-1.72) | | (0.92-1.30) | |
| Do not know | 1.87* | 1.60 | 2.25** | 1.60 | 1.80* | | 1.87* | |
|  | (1.01-3.44) | (0.78-3.17) | (1.36-3.72) | (0.96-2.66) | (1.04-3.09) | | (1.16-3.03) | |
| Parents’ university/ college education |  |  |  |  |  | |  | |
| At least one parent (ref.) | 1.00 | 1.00 | 1.00 | 1.00 | 1.00 | | 1.00 | |
| No one | 0.99 | 1.05 | 0.98 | 1.07 | 1.15 | | 1.09 | |
|  | (0.78-1.27) | (0.81-1.36) | (0.79-1.23) | (0.86-1.33) | (0.93-1.42) | | (0.87-1.35) | |
| Do not know | 0.91 | 0.91 | 0.99 | 1.04 | 1.18* | | 0.98 | |
|  | (0.75-1.11) | (0.74-1.12) | (0.84-1.16) | (0.89-1.23) | (1.00-1.39) | | (0.83-1.15) | |

*p<0.05, **p<0.01, ***p<0.001

Table A7. Adolescents’ psychosomatic complaints (more than weekly) regressed on perceived parental alcohol problems (CAST-6, cutoff ≥3). Odds ratios (OR) and 95% confidence intervals (95% CI) from binary logistic regression models with robust standard errors. Grade 11 only (n= 4,140)

|  | Headache | Stomach ache | Depressed and down | Difficulty in falling asleep | | Slept poorly at night | | Psychosomatic complaints (≥2) |
| --- | --- | --- | --- | --- | --- | --- | --- | --- |
|  | OR | OR | OR | OR | OR | | OR | |
|  | (95% CI) | (95% CI) | (95% CI) | (95% CI) | (95% CI) | | (95% CI) | |
| Perceived parental alcohol problems (CAST-6, cutoff ≥3) |  |  |  |  |  | |  | |
| No (ref.) | 1.00 | 1.00 | 1.00 | 1.00 | 1.00 | | 1.00 | |
| Yes | 1.46** | 1.49*** | 1.89*** | 1.47*** | 1.62*** | | 1.73*** | |
|  | (1.18-1.81) | (1.19-1.87) | (1.57-2.27) | (1.22-1.78) | (1.36-1.93) | | (1.44-2.07) | |
| Gender |  |  |  |  |  | |  | |
| Boys (ref.) | 1.00 | 1.00 | 1.00 | 1.00 | 1.00 | | 1.00 | |
| Girls | 3.80*** | 5.09*** | 3.08*** | 1.40*** | 1.47*** | | 2.28*** | |
|  | (3.14-4.58) | (4.07-6.37) | (2.63-3.60) | (1.19-1.63) | (1.26-1.70) | | (1.98-2.63) | |
| Other | 2.67** | 4.58*** | 3.71*** | 2.04* | 2.07* | | 2.06* | |
|  | (1.31-5.43) | (2.06-10.21) | (1.83-7.53) | (1.12-3.73) | (1.07-4.01) | | (1.08-3.93) | |
| Parents’ country of birth |  |  |  |  |  | |  | |
| At least one in Sweden (ref). | 1.00 | 1.00 | 1.00 | 1.00 | 1.00 | | 1.00 | |
| Two abroad | 1.51*** | 1.31* | 1.24* | 1.45*** | 1.95*** | | 1.62*** | |
|  | (1.20-1.90) | (1.01-1.70) | (1.01-1.51) | (1.20-1.75) | (1.61-2.35) | | (1.34-1.96 | |
| Do not know | 1.93 | 1.49 | 1.78 | 1.97* | 2.57** | | 2.55** | |
|  | (0.85-4.41) | (0.56-3.93) | (0.87-3.65) | (1.05-3.68) | (1.37-4.80) | | (1.37-4.75) | |
| Parents’ university/ college education |  |  |  |  |  | |  | |
| At least one parent (ref.) | 1.00 | 1.00 | 1.00 | 1.00 | 1.00 | | 1.00 | |
| No one | 1.01 | 0.92 | 0.79* | 1.08 | 1.01 | | 0.94 | |
|  | (0.80-1.28) | (0.73-1.17) | (0.65-0.96) | (0.90-1.31) | (0.85-1.20) | | (0.78-1.12) | |
| Do not know | 1.06 | 0.93 | 1.06 | 1.38** | 1.11 | | 1.14 | |
|  | (0.83-1.36) | (0.71-1.22) | (0.86-1.29) | (1.13-1.69) | (0.90-1.37) | | (0.93-1.39) | |

*p<0.05, **p<0.01, ***p<0.001
